# Phytoremediation of Benzophenone and Bisphenol A by Glycosylation with Immobilized Plant Cells

**DOI:** 10.4137/EHI.S897

**Published:** 2009-04-06

**Authors:** Kei Shimoda, Hatsuyuki Hamada, Hiroki Hamada

**Affiliations:** 1 Department of Pharmacology and Therapeutics, Faculty of Medicine, Oita University, 1-1 Hasama-machi, Oita 879-5593, Japan; 2 National Institute of Fitness and Sports in Kanoya, 1 Shiromizu-cho, Kagoshima 891-2390, Japan; 3 Department of Life Science, Faculty of Science, Okayama University of Science, 1-1 Ridai-cho, Okayama 700-0005, Japan

**Keywords:** glycosylation, benzophenone, bisphenol A, β-glycoside, Nicotiana tabacum, immobilized cells

## Abstract

Benzophenone and bisphenol A are environmental pollutions, which have been listed among “chemicals suspected of having endocrine disrupting effects” by the World Wildlife Fund, the National Institute of Environmental Health Sciences in the USA and the Japanese Environment Agency. The cultured cells of *Nicotiana tabacum* glycosylated benzophenone to three glycosides, 4-*O*-β-D-glucopyranosylbenzophenone (9%), diphenylmethyl β-D-glucopyranoside (14%), and diphenylmethyl 6-*O*-(β-D-glucopyranosyl)-β-D-glucopyranoside (12%) after 48 h incubation. On the other hand, incubation of benzophenone with immobilized cells of *N. tabacum* in sodium alginate gel gave products in higher yields, i.e. the yields of 4-*O*-β-D-glucopyranosylbenzophenone, diphenylmethyl β-D-glucopyranoside, and diphenylmethyl 6-*O*-(β-D-glucopyranosyl)-β-D-glucopyranoside were 15, 27, and 22%, respectively. Bisphenol A was converted into three glycosides, 2,2-bis(4-β-D-glucopyranosyloxyphenyl)propane (16%), 2-(4-β-D-glucopyranosyloxy-3-hydroxyphenyl)-2-(4-β-D-glucopyranosyloxyphenyl) propane (8%), and 2-(3-β-D-glucopyranosyloxy-4-hydroxyphenyl)-2-(4-β-D-glucopyranosyloxyphenyl)propane (5%). Also the use of immobilized *N. tabacum* cells improved the yield of products; the glycosylation of bisphenol A with immobilized *N. tabacum* gave 2,2-bis(4-β-D-glucopyranosyloxyphenyl)propane (24%), 2-(4-β-D-glucopyranosyloxy-3-hydroxyphenyl)-2-(4-β-D-glucopyranosyloxyphenyl) propane (15%), and 2-(3-β-D-glucopyranosyloxy-4-hydroxyphenyl)-2-(4-β-D-glucopyranosyloxyphenyl)propane (11%).

## Introduction

Use of plant cell culture is a new approach to biotransformation research. Furthermore, biological potential of plant cell culture to biotransform exogenously added toxic compounds has attracted much attention in pollution control.^1^–^5^ The biotransformation reactions catalyzed by cultured plant cells include oxidation, reduction, hydroxylation, esterification, hydrolysis, methylation, glycosylation, and isomerization. Hydroxylation and glycosylation are particularly efficient procedures for the phytoremediation of environmental pollution.^6^,^7^

Benzophenone and bisphenol A are widely used as the starting material for the production of polyacrylates, ether resins, phenol resins, insecticides, agricultural chemicals, pharmaceuticals, and coatings, and are released as pollutants and toxic compounds into the environment.^8^ Recently, it has been reported that benzophenone and bisphenol A exhibited estrogenic activity in bioassays.^9^ They have been listed among “chemicals suspected of having endocrine disrupting effects” by the World Wildlife Fund, the National Institute of Environmental Health Sciences in the U.S.A. and the Japanese Environment Agency. Up to now little attention has been paid to the biotransformation of such endocrine-disrupting chemicals with cultured plant cells. We now report the biotransformation of benzophenone and bisphenol A to the corresponding glycosides, which are accumulated in the cells to lower the concentration of the substrates in the medium, by immobilized cells of *Nicotiana tabacum* in sodium alginate gel.

## Material and Methods

### General

Benzophenone and bisphenol A were purchased from Aldrich Chemical Co. The ^1^H and ^13^C NMR, H-H COSY, C-H COSY, and HMBC spectra were recorded using a Varian XL-400 spectrometer in CD_3_OD solution. The chemical shifts were expressed in *δ* (ppm) referring to TMS. The FABMS spectra were measured using a JEOL MStation JMS-700 spectrometer. Cultured plant cells of *N. tabacum* have been cultivated over 20 years in our laboratory and subcultured in 300 ml conical flasks containing Murashige and Skoog’s medium (100 ml, pH 5.7) on a rotary shaker (120 rpm) at 25 °C in the dark for every 3–5 weeks.

### Biotransformation of benzophenone and bisphenol a by the cultured cells of *N. tabacum*

To the 500 ml flask containing the suspension cultured cells of *N. tabacum* was added 15 mg of substrate. The cultures were incubated at 25 °C for 48 h on a rotary shaker (120 rpm) in the dark. After the incubation period, the cells and medium were separated by filtration with suction. The filtered medium was extracted with EtOAc. The cells were extracted (x3) by homogenization with MeOH, and the MeOH fraction was concentrated and partitioned between H_2_O and EtOAc. The EtOAc fractions were combined, concentrated, and analyzed by HPLC [column: CRESTPAK C18S column (150 × 4.6 mm); solvent: Dioxane:H_2_O (1:9, v/v); detection: UV (280 nm); flow rate: 1.0 ml/min]. The H_2_O fraction was applied to a Dianion HP-20 column and the column was washed with H_2_O followed by elution with MeOH. The MeOH eluate was subjected to preparative HPLC [column: CAPCELLPAK R&D C18 column (250 × 30 mm); solvent: MeOH:H_2_O (9:11, v/v); detection: UV (280 nm); flow rate: 1.0 ml/min] to give products. The yield of the products was determined on the basis of the peak area from HPLC and expressed as a relative percentage to the total amount of the whole reaction products extracted.

Spectral data of products: 4-*O*-β-D-glucopyranosylbenzophenone (**2**): *m*/*z* 361 [M+H]^+^; ^1^H NMR (400 MHz, CD_3_OD): *δ* 3.73 (1H, dd, *J* = 12.0, 5.6 Hz, H-6b’), 3.92 (1H, dd, *J* = 12.0, 2.0 Hz, H-6a’), 5.05 (1H, d, *J* = 7.2 Hz, H-1’), 7.20 (2H, d, *J* = 8.8 Hz, H-3,5), 7.50 (2H, t, *J* = 8.0 Hz, H-10, 12), 7.60 (1H, t, *J* = 7.6 Hz, H-11), 7.70 (2H, d, *J* = 8.4 Hz, H-9, 13), 7.77 (2H, d, *J* = 8.8 Hz, H-2, 6). Diphenylmethyl β-D-glucopyranoside (**3**): FABMS: *m*/*z* 369 [M+Na]^+^; ^1^H NMR (400 MHz, CD_3_OD): *δ* 3.67 (1H, dd, *J* = 12.2, 6.4 Hz, H-6b’), 3.88 (1H, dd, *J* = 12.2, 2.0 Hz, H-6a’), 4.27 (1H, d, *J* = 7.2 Hz, H-1’), 6.05 (1H, s, H-7), 7.18 (1H, t, *J* = 7.6 Hz, H-4), 7.23 (3H, t, *J* = 7.6 Hz, H-3, 5, 11), 7.33 (2H, t, *J* = 8.0 Hz, H-10, 12), 7.39 (2H, d, *J* = 7.6 Hz, H-2, 6), 7.47 (2H, d, *J* = 7.2 Hz, H-9, 13). Diphenylmethyl 6-*O*-(β-D-glucopyranosyl)-β-D-glucopyranoside (**4**): FABMS: *m*/*z* 531 [M+Na]^+^; ^1^H NMR (400 MHz, CD_3_OD): *δ* 3.66 (1H, dd, *J* = 12.0, 5.6 Hz, H-6b”), 3.81 (1H, dd, *J* = 11.6, 5.2 Hz, H-6b’), 3.86 (1H, dd, *J* = 11.6, 2.0 Hz, H-6a”), 4.15 (1H, d, *J* = 11.2 Hz, H-6a’), 4.30 (1H, d, *J* = 7.6 Hz, H-1”), 4.45 (1H, d, *J* = 7.6 Hz, H-1’), 6.05 (1H, s, H-7), 7.18 (1H, t, *J* = 8.0 Hz, H-4), 7.26 (3H, t, *J* = 7.6 Hz, H-3, 5, 11), 7.33 (2H, t, *J* = 8.0 Hz, H-10, 12), 7.40 (2H, d, *J* = 7.6 Hz, H-2, 6), 7.51 (2H, d, *J* = 7.2 Hz, H-9, 13). 2,2-Bis(4-β-D-glucopyranosyloxyphenyl)propane (**6**): FABMS: *m*/*z* 575 [M+Na]^+^; ^1^H NMR (400 MHz, CD_3_OD): *δ* 1.61 (6H, s, H-1, 3), 4.87 (2H, d, *J* = 8.0 Hz, H-1’, 1”), 6.98 (4H, d, *J* = 8.5 Hz, H-6, 8, 12, 14), 7.13 (4H, d, *J* = 8.5 Hz, H-5, 9, 11, 15). 2-(4-β-D-Glucopyranosyloxy-3-hydroxyphenyl)-2-(4-β-D-glucopyranosyloxyphenyl)propane (**7**): FABMS: *m*/*z* 591 [M+Na]^+^; ^1^H NMR (400 MHz, CD_3_OD): *δ* 1.60 (6H, s, H-1, 3), 4.70 (1H, d, *J* = 7.8 Hz, H-1’), 4.86 (1H, d, *J* = 8.0 Hz, H-1”), 6.66 (1H, dd, *J* = 8.0, 2.0 Hz, H-9), 6.68 (1H, d, *J* = 2.0 Hz, H-5), 6.98 (2H, d, *J* = 8.5 Hz, H-12, 14), 7.08 (1H, d, *J* = 8.0 Hz, H-8), 7.14 (2H, d, *J* = 8.5 Hz, H-11, 15). 2-(3-β-D-Glucopyranosyloxy-4-hydroxyphenyl)-2-(4-β-D-glucopyranosyloxyphenyl)propane (**8**): FABMS: *m*/*z* 591 [M+Na]^+^; ^1^H NMR (400 MHz, CD_3_OD): *δ* 1.60 (6H, s, H-1, 3), 4.60 (1H, d, *J* = 7.6 Hz, H-1”), 4.88 (1H, d, *J* = 8.0 Hz, H-1’), 6.72 (1H, d, *J* = 8.5 Hz, H-8), 6.81 (1H, dd, *J* = 8.5, 2.0 Hz, H-9), 6.90 (1H, d, *J* = 2.0 Hz, H-5), 7.00 (2H, d, *J* = 8.5 Hz, H-12, 14), 7.16 (2H, d, *J* = 8.5 Hz, H-11, 15).

### Preparation of immobilized cells of *N. tabacum* in sodium alginate gel

Sodium alginate (2%) was suspended in water (500 ml), which was autoclaved at 120 °C for 30 min. Cultured cells of *N. tabacum* (100 g) in the stationary growth phase were added to this solution and the mixture was stirred for 2 h until it became homogeneous. The suspension was added dropwise from a dropping funnel with a glass tube into a 5% CaCl_2_ solution (1 L) with stirring to form pieces of spherical sodium alginate gel with 5 mm diameter immediately. After washing with water, immobilized *N. tabacum* cells were used for biotransformation of benzophenone and bisphenol A.

### Biotransformation of benzophenone and bisphenol a by immobilized cells of *N. tabacum* in sodium alginate gel

To 150 ml of the immobilized *N. tabacum* cells which included 30 g of *N. tabacum* cells with 100 ml of MS medium containing 3% sucrose in a 500 ml conical flask was added 15 mg substrate. The flask was stirred at 25 °C for 48 h on a rotary shaker (120 rpm) in the dark. Products were extracted from the cells and were purified by the same method as described above.

## Results

**Scheme 1.**
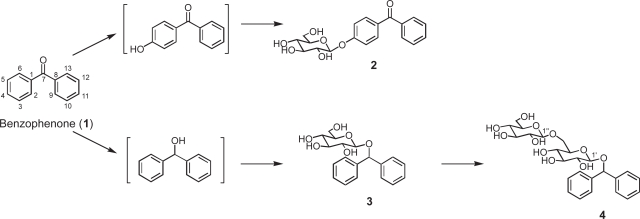
Biotransformation pathway of benzophenone (**1**) by cultured and immobilized plant cells of *N. tabacum*.

Biotransformation products were isolated from the MeOH extracts of the cultured cells of *N. tabacum*, which had been treated with benzophenone (**1**) for 48 h. No products were observed in the medium. Three potentially glycosylated products **2**–**4** were obtained, and no additional conversion products such as hydroxylated and reduced products were observed in spite of the careful HPLC analyses. The chemical structures of the products were determined on the basis of their FABMS, ^1^H and ^13^C NMR, H-H COSY, C-H COSY, and HMBC spectra as 4-*O*-β-D-glucopyranosylbenzophenone (**2**, 9%), diphenylmethyl β-D-glucopyranoside (**3**, 14%), and diphenylmethyl 6-*O*-(β-D-glucopyranosyl)-β-D-glucopyranoside (**4**, 12%) (Scheme 1).

**Figure 1.**
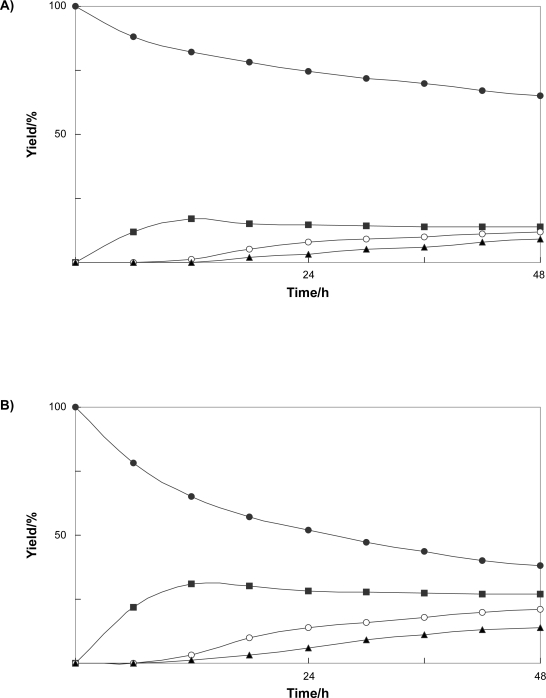
Time course of the biotransformation of benzophenone (**1**) (**A**) by cultured cells and (**B**) by immobilized cells of *N. tabacum*. Yields of **1** (•), **2** (▴), **3** (▪), and **4** (○) are plotted.

In order to examine the ability of cultured *N. tabacum* cells to biotransform benzophenone (**1**), the time course in the conversion of **1** was followed. Figure 1A showed that benzophenone (**1**) was converted into **3** at early stage of incubation, and that the product **4** was accumulated in the cells after 24 h incubation. This result indicated that formation of disaccharide **4** occurred following to that of glucoside **3**.

**Figure 2.**
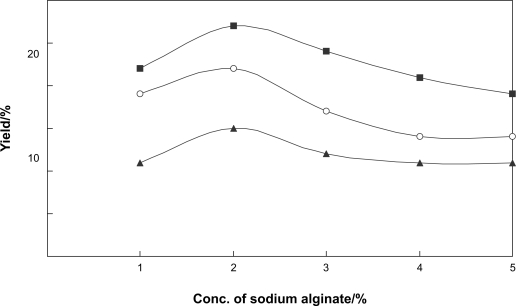
Effects of sodium alginate concentration on the glycosylation activity of the immobilized cells of *N. tabacum*. Yields of **2** (▴), **3** (▪), and **4** (○) are plotted.

Next, the conversion of benzophenone (**1**) by immobilized *N. tabacum* cells with sodium alginate was investigated. To examine the effect of sodium alginate concentration on the glycosylation of benzophenone (**1**), the cells were immobilized with sodium alginate at final concentrations of 1, 2, 3, 4, and 5%. Figure 2 shows that the glycoside formation was enhanced at 2% sodium alginate concentration. The immobilized *N. tabacum* cells, which had been prepared at 2% sodium alginate concentration, were used for biotransformation experiments. After 48 h incubation of benzophenone (**1**), 4-*O*-β-D-glucopyranosylbenzophenone (**2**, 15%), diphenylmethyl β-D-glucopyranoside (**3**, 27%), and diphenylmethyl 6-*O*-(β-D-glucopyranosyl)-β-D-glucopyranoside (**4**, 22%) were obtained in higher yields in comparison with the case of the biotransformation using normal cells (Figure. 1B). The time course experiment also showed that the conversion rate of glycosylation of benzophenone (**1**) was higher than that in the case of the time course of the biotransformation using normal cells.

**Figure 3.**
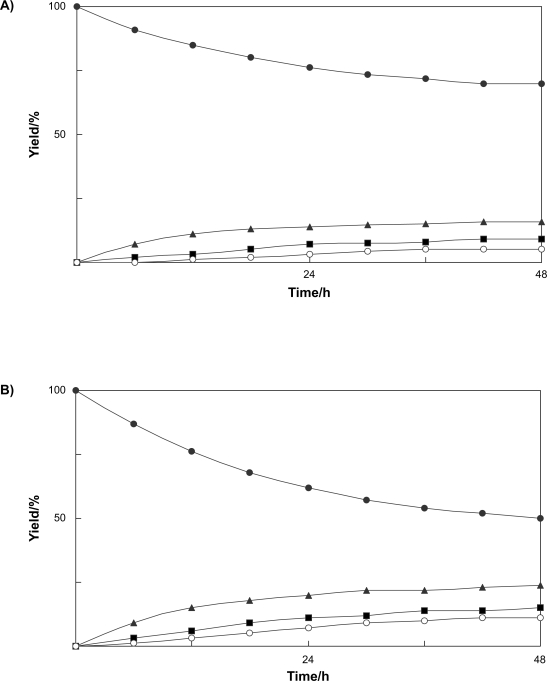
Time course of the biotransformation of bisphenol A (**5**) (**A**) by cultured cells and (**B**) by immobilized cells of *N. tabacum*. Yields of **5** (•), **6** (▴), **7** (▪), and **8** (○) are plotted.

**Scheme 2.**
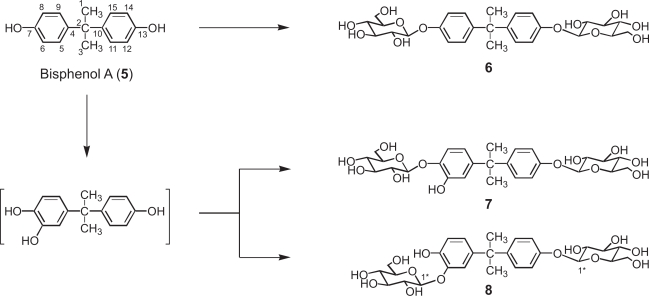
Biotransformation pathway of bisphenol A (**5**) by cultured and immobilized plant cells of *N. tabacum*.

On the other hand, bisphenol 2,2-bis(4-β-D-glucopyranosyloxyphenyl) propane (**6**, 16%), 2-(4-β-D-glucopyranosyloxy-3-hydroxyphenyl)-2-(4-β-D-glucopyranosyloxyphenyl) propane (**7**, 8%), and 2-(3-β-D-glucopyranosyloxy-4-hydroxyphenyl)-2-(4-β-D-glucopyranosyloxyphenyl)propane (**8**, 5%) were isolated from cultured *N. tabacum* cells treated with bisphenol A (**5**) after 48 h-incubation (Scheme 2). The time course in the conversion of **5** showed that **6** predominantly accumulated rather than **7** and **8** (Figure 3A). Use of immobilized *N. tabacum* cells improved the yield of products, that is, the glycosylation of bisphenol A (**5**) with immobilized *N. tabacum* cells gave 2,2-bis(4-β-D-glucopyranosyloxyphenyl)propane (**6**, 24%), 2-(4-β-D-glucopyranosyloxy-3-hydroxyphenyl)-2-(4-β-D-glucopyranosyloxyphenyl) propane (**7**, 15%), and 2-(3-β-D-glucopyranosyloxy-4-hydroxyphenyl)-2-(4-β-D-glucopyranosyloxyphenyl) propane (**8**, 11%) (Fig. 3B).

## Discussion

This study demonstrated that the cultured plant cells of *N. tabacum* can convert benzophenone and bisphenol A into three glycosides, respectively. In the time course experiment, it was found that reduction of benzophenone first occurred, followed by glycosylation of the newly generated hydroxyl group. The yield of glycosides produced from benzophenone by immobilized *N. tabacum* cells in sodium alginate gel was improved in comparison with the case of biotransformation by cultured *N. tabacum* cells. Total yield of glycosides of benzophenone produced by immobilized *N. tabacum* cells was about 1.8 times higher than that of products obtained in normal biotransformation experiment. The use of immobilized cells of *N. tabacum* for conversion of bisphenol A also enhanced the yield of the products, that is, the total yield of glycosides was about 1.7 fold higher than that of products obtained in normal biotransformation experiment.

The results of this experiment revealed that plant cells of *N. tabacum* can incorporate the endocrine-disrupting chemicals such as benzophe-none and bisphenol A to biotransform into glycosides, and can lower their concentration in the medium. The immobilized cells of *N. tabacum* would be useful to detox industrial by-products, i.e. benzophenone and bisphenol A, to stable glycosides. This procedure is a simple operation and is environmentally friendly. Further studies on the enzymes which catalyze the glycosylation of benzophenone and bisphenol A are now in progress.
